# Feasibility of classic massage for Swedish nurses: a mixed methods pre-post-test design study

**DOI:** 10.1186/s12906-026-05356-9

**Published:** 2026-03-26

**Authors:** Mats Westas, Lena Ekström, Jan Sandqvist, Charlotte Wåhlin, Gerhard Andersson, Peter Johansson

**Affiliations:** 1 Department of Health, Medicine, and Caring Sciences, Linköping University, Norrköping, Sweden; 2 Department of Internal Medicine, Linköping University, Norrköping, Sweden; 3 Department of Health, Medicine and Caring Sciences, Division of Prevention, Rehabilitation and Community Medicine, Unit of Occupational Therapy, Linköping University, Norrköping, Sweden; 4 Occupational and Environmental Medicine Centre, Department of Health, Medicine and Caring Sciences, Unit of Clinical Medicine, Linköping University, Linköping, Sweden; 5 Department of Behavioural Sciences and Learning, Linköping University, Linköping, Sweden; 6 Department of Biomedical and Clinical Sciences, Linköping University, Linköping, Sweden

**Keywords:** Feasibility Studies, Health Personnel, Massage Therapy, Stress, Psychological, Mental health

## Abstract

**Background:**

Nurses face demanding work linked to poor mental and physical health. Evidence for Swedish classic massage in this context is limited to short-term trials with passive comparators. Before a randomised controlled trial, feasibility should be evaluated. Guided by Bowen’s feasibility framework, we examined a 12-month, monthly Swedish classic massage programme for nurses, focusing on limited efficacy (stress, mental health, sleep, physical activity, HR-QoL, pain, work ability) and acceptability (recruitment, adherence, satisfaction).

**Methods:**

An embedded mixed-methods, one-group pre–post design was used. Quantitative outcomes were assessed at baseline, 6 months, and 12 months. Limited efficacy was defined as ≥ 3 target domains with effect sizes ≥ 0.20 at 6 or 12 months. Acceptability was appraised using predefined traffic light criteria (Green, Amber, Red) for recruitment, adherence, and satisfaction. Qualitative data from open-ended questions at 12 months explored participants’ experiences.

**Results:**

Twenty-five nurses participated (84% completed follow-up). Recruitment reached 50% of the target (Amber), while adherence and satisfaction met Green thresholds. According to predefined criteria, limited efficacy was achieved, with ≥ 3 target domains exceeding the 0.20 threshold at both 6 and 12 months. Domains contributing to this signal included stress, HR-QoL, work ability, and mental health, with many changes evident by 6 months. Depressive symptoms and exhaustion continued to improve to 12 months, while sleep and sustained pain relief contributed only partially to the limited efficacy signal. Integration of quantitative and qualitative data indicated that perceived reductions in stress, increased emotional balance, and greater capacity to manage work demands aligned with measured outcomes.

**Conclusion:**

This feasibility study met predefined acceptability and limited efficacy criteria, except for recruitment. Progression to a larger trial in this format is not warranted; however, modifications such as extended recruitment, repeated invitations, and preference-based designs may improve feasibility. Domains with weaker or inconsistent change may require targeted co-interventions in a future evaluation.

**Trial registration:**

ClinicalTrials.gov NCT05555082

## Key points

• Offering monthly classic massage for nurses was feasible to deliver and was well received, with high adherence and satisfaction. Recruitment was lower than planned, indicating the need for changes to attract more participants.

• The study met its predefined criteria, with several outcomes such as stress, quality of life, work ability, and mental health showing consistent positive signals at both 6 and 12 months. Some areas, like sleep and lasting pain relief, showed weaker or less consistent signals.

• A larger trial in this exact form is not advised. Future studies should test adjusted recruitment approaches (e.g., longer recruitment periods, reminders, and offering choice between interventions) and add targeted components to address outcomes that were less responsive.

## Background

Nurses working on hospital wards face demanding working conditions that are linked to adverse mental and physical health outcomes. Nurse-specific studies report substantial levels of stress, anxiety, sleep problems, and depressive symptoms [1–3]. Broader cross sector evidence including health care works (HCW) shows that high job demands are associated with poorer health and wellbeing [4]. In this study, our population is nurses; we use HCWs only when referring to broader workforce data or policies that include nurses.

Physically, these mental health challenges commonly lead to conditions like high blood pressure, diabetes, and an increased risk of cardiovascular disease [4]. For example, psychological stress can dysregulate the hypothalamic-pituitary-adrenal (HPA) axis, and lead to increased cortisol levels and systemic inflammation, which may contribute to metabolic disturbances and increase the risk for cardiovascular disease [5]. Another prevalent issue among HCW is musculoskeletal pain, with studies indicating that 25–60% of HCWs experience pain [6, 7]. Research also suggests that mental health issues can heighten the perception of pain, which, in turn, can amplify workload and stress [8].

The demanding workload on hospital wards contributes to high turnover among nurses [1, 9], which, in turn, can result in reduced productivity and increased staff shortage and place additional strain on care delivery. These dynamics are associated with lower care quality and heightened risk to patient safety during treatment [9, 10]. High turnover rates also increases workload on the remaining staff, elevating stress and reducing job satisfaction [11]. In Sweden, health and social care workers (HCWs. including nurses) have the highest rates of sickness leave, with mental illness and musculoskeletal pain being the most common causes [12]. Sustainable improvement will require action at both the organizational and individual levels.

In line with recommendations from the Swedish Work Environment Authority for a combined approach, we examine classic massage as an individual-level strategy that could be feasible within broader workplace health efforts. Although massage can be perceived as relatively costly due to its one-to-one format, it is already commonly offered through employer-sponsored wellness allowances in Swedish healthcare; thus, feasibility may depend as much on acceptability, accessibility, and practical delivery as on cost. If shown to be beneficial, classic massage could help reduce stress-related problems and absenteeism over time. Against this background, evaluating interventions that address stress, mental health, and musculoskeletal symptoms among nurses is essential not only to understand potential benefits but also to assess their contribution to workforce sustainability and healthcare system resilience [13].

In this study, “classic massage” refers to Swedish massage techniques involving kneading, gliding, and tapping of soft tissues, primarily muscles [14]. Across two literature reviews of synthesizing studies of wellness interventions in HCWs (15 and 33 studies respectively) [15, 16], only Zhang et al. [15] identified three trials of classic massage in their review, all of which trials were short duration randomized trials conducted in nurse samples ( Bost et al. [17]; Nazari et al. [18] and Mahdizadeh et al. [19]). These programs were brief, 15 min once weekly for 5 weeks [17] or 25 min twice weekly for 4 weeks [18, 19], used passive comparators (i.e., no intervention) had minimal or no long-term follow-up and only reporting effects on stress reduction. They were also conducted in contexts unlike Swedish hospital wards (two trials in Iran [18, 19]), which limits direct comparability. Because prior evaluations were short-term and relied on passive comparators, we hypothesized that broader symptom domains such as stress, anxiety, low mood, sleep disturbance, and perceived work ability may require sustained, low-intensity exposure to detect within-group change. Accordingly, rather than proceeding directly to a randomized trial, we first sought to evaluate feasibility of delivering classic massage monthly for 12 months among Swedish nurses. Guided by Bowen et al. (2009) framework of feasibility [20], our aim was to: (1) conduct limited efficacy testing of massage of changes in stress, mental health, physical activity, health-related quality of life (HR-QoL), including pain, and work ability; and (2) assess acceptability of using quantitative and qualitative data, focusing on recruitment, adherence and participant satisfaction and experiences.

## Methods

### Study design

This study used an embedded mixed methods pre-post design, where qualitative data were collected at the final follow-up to complement and help interpret quantitative finding [21]. A mixed-methods approach combines quantitative and qualitative data to explore a phenomenon more comprehensively, allowing statistical trends (quantitative) to be explained through qualitative insights and qualitative findings to complement or expand quantitative results [21]. The embedded design is flexible and adaptable, making it valuable for complex research questions where both numerical and narrative insights are needed. Quantitative data were prioritized to appraise limited efficacy, defined under Bowen’s feasibility framework as a preliminary within group assessment of the direction and magnitude of change, and to assess acceptability indicators including recruitment, adherence, and participant satisfaction [20]. Qualitative data were collected at 12 months to deepen understanding of participants’ experiences [21]. Integration occurred during analysis by comparing qualitative themes with quantitative outcomes to examine convergence and divergence, with details provided in the Results and the Discussion.

### Participants

While the literature reviewed in the introduction refers to (HCW) broadly, our sample was limited to registered and assistant nurses. This focus reflects the reality of the inpatient ward setting, where these professionals are most exposed to physically and emotionally demanding conditions, including shift work and close patient contact. Thus, registered and assistant nurses working in a medical ward at a Swedish hospital were eligible to participate in the study. To be included, participants needed to work in patient care at the ward for at least 50% of full-time hours. Approximately 130 nurses were estimated to meet the eligibility criteria, and we planned to recruit 50 nurses. The sample size was pragmatically based on operational capacity and resource availability, for example available funding only allowing us to offer a limited number of massage sessions. In addition, the number was considered reasonable to assess feasibility parameters such as recruitment yield, adherence, and acceptability, in line with Bowen et al. (2009) [20]. While not statistically powered, the target was selected to support interpretation of feasibility outcomes. Recruitment was scheduled for a one-month period (November–December 2022) before the planned start of the intervention in January 2023. To raise awareness ahead of this short recruitment window, information about the upcoming study was shared with staff during routine staff meetings prior to the formal registration period. Recruitment was then carried out through an advertisement on the clinic’s website and an email sent to all healthcare staff at the clinic. The recruitment email was directed specifically to the clinic’s nursing staff, in accordance with internal mailing list structures. Other professional groups, such as physicians, physiotherapists, or social workers, were not contacted and were not eligible to participate. Both the advertisement and the email provided detailed information about the study, including the maximum number of participants (50), and included a link to a registration form for those interested. The form collected participants’ names, phone numbers, and email addresses.

Individuals who registered and meet the inclusion criteria were emailed and invited to meet with the research nurse. During the appointment, participants had the opportunity to ask the nurse additional questions about the study and were asked to provide written consent for participation. At the visit baseline measures were obtained, including blood pressure, height, and weight, together with the baseline questionnaires.

### The intervention

The participants received classic massage in 30-minute sessions approximately once a month over the course of one year (i.e., 2023). This duration was based on the standard hands-on treatment time offered by the certified massage therapists contracted for the study. Massage sessions were scheduled outside of working hours in agreement between each participant and the therapist and took place in a quiet and private room at a massage clinic to support relaxation.

Two certified massage therapists were engaged for the study, and participants were evenly divided between them. Each participant received all their sessions from the same therapist to promote continuity and individual adaptation of the treatment. Both therapists were certified in classic Swedish massage and had 15 and 21 years of experience operating private massage practices. The treatment followed the principles of classic massage, including kneading, gliding, and tapping techniques, and primarily targeted areas commonly affected by work-related strain, such as the back, shoulders, and neck. The depth and focus of the massage were tailored to individual needs through ongoing dialogue with each participant. Because ward operations and summer vacations were expected to potentially reduce availability in June and July, the protocol allowed rescheduling across adjacent months; no blanket pause was planned. The target total exposure per participant was 10 to 12 sessions within twelve months, depending on individual scheduling. No exclusion criteria were set based on exact month-to-month spacing.

### Data collection

Data was collected at three time points: baseline, 6 months, and at the end of the massage intervention after 12 months. Baseline data were collected at the initial appointment with the research nurse. Prior to this appointment, participants received a package of questionnaires to complete at home, which they returned during the visit. This procedure allowed participants time to reflect on the questions while ensuring that the completed forms could be checked for completeness or clarified during the appointment. These questionnaires covered background variables and outcome variables of interest (i.e., stress, mental health, sleeping problems, physical activity, HR-QoL and work ability). Participants were provided with a stamped envelope to return the completed questionnaires, either by handing them to the research nurse or by mailing them to the project leaders at the clinic, PJ or LE. At the 6-month follow-up, participants completed the same set of questionnaires as at baseline which included questions about the number of massage sessions they attended, as well as questions about stress, mental health, sleep problems, HR-QoL, and work ability. At the end of the intervention at 12 months (December 2023–January 2024), participants again completed the same set of questionnaires, along with additional items on physical activity, satisfaction, and open-ended questions. On this occasion, they also rated their physical activity and scored their satisfaction with the massage on a scale, as well as responded to open-ended questions, allowing for the collection of both quantitative and qualitative data at 12 months-follow-up.

## Feasibility outcomes

### Limited efficacy

The quantitative outcome measures were collected to explore such early signals of effect, assess the relevance and sensitivity of the instruments used, and inform the design of a future fully powered trial. This does not imply low effectiveness, but rather a structured exploration of whether further testing is warranted [20].

#### Quantitative data collection.

##### Stress

Stress levels were evaluated using the Perceived Stress Scale-10 (PSS-10) [22]. The questionnaire consists of 10 items that assess how unpredictable, uncontrollable, and overloaded respondents find their lives. Participants rate each item on a 5-point Likert scale, ranging from 0 (never) to 4 (very often), reflecting their feelings and thoughts during the past month. The scores for the PSS-10 range from 0 to 40, with higher scores indicating higher perceived stress. The PSS-10 has been validated in Swedish and is considered a reliable indicator of perceived stress [23].

##### Mental health

We employed three different questionnaires to assess various aspects of mental health: the Patient Health Questionnaire-9 (PHQ-9) [24] for depressive symptoms, the General Anxiety Disorder Scale-9 (GAD-7) [25] for anxiety, and the Karolinska Exhaustion Disorder Scale (KEDS) [26] for exhaustion.

PHQ-9 consists of nine questions aligned with the diagnostic criteria for major depressive disorder in the DSM-IV [24]. Each question is scored on a scale from 0 (not at all) to 3 (nearly every day), considering the frequency of symptoms over the past two weeks. The total score, ranging from 0 to 27, helps gauge the severity of depression, with higher scores indicating more severe depressive symptoms. The cut-off scores for PHQ-9 are as follows: Minimal depression ranges from 0 to 4, mild depression from 5 to 9, moderate depression from 10 to 14, moderately severe depression from 15 to 19, and severe depression from 20 to 27. A ≥ 20% reduction from baseline is typically used to indicate a clinically meaningful improvement in depressive symptoms [24].

GAD-7 [25] comprises seven questions, each rated on a scale from 0 (not at all) to 3 (nearly every day), reflecting the frequency of anxiety symptoms over the past two weeks. The total score ranges from 0 to 21, with higher scores indicating greater anxiety severity. The cut-off scores for GAD-7 are as follows: minimal anxiety ranges from 0 to 4, mild anxiety from 5 to 9, moderate anxiety from 10 to 14, and severe anxiety from 15 to 21. A 4-point reduction has been suggested as the threshold for minimum clinically important difference (MCID) [27].

KEDS [26] consists of nine questions that assess exhaustion symptoms over the past two weeks. Each question is scored on a scale from 0 (never) to 6 (always), with a total score ranging from 0 to 54. Higher scores indicate greater difficulty with exhaustion. The cut-off scores for KEDS are as follows: mild exhaustion ranges from 0 to 18, moderate exhaustion from 19 to 29, and severe exhaustion from 30 to 54.

##### Sleep problems

The Insomnia Severity Index (ISI) [28] assessed the severity of both nighttime and daytime components of insomnia. It consists of seven items that evaluate the nature, severity, and impact of insomnia over the past two weeks. Each item is rated on a 5-point Likert scale ranging from 0 (no problem) to 4 (very severe problem), with the total score ranging from 0 to 28. Higher scores on the ISI indicate greater severity of insomnia symptoms. The cut-off scores for interpreting the ISI are as follows: scores from 0 to 7 indicate no clinically significant insomnia; scores from 8 to 14 suggest subthreshold insomnia; scores from 15 to 21 reflect clinical insomnia of moderate severity; and scores from 22 to 28 indicate severe clinical insomnia. For ISI, two MCID thresholds have been proposed based on different outcome anchors. A 6-point reduction is commonly used to indicate clinically meaningful improvement in individuals with primary insomnia [29]. However, smaller changes around 2.5 points have also been associated with meaningful improvements in functioning and mood in non-clinical populations [29]. In this study, we report the proportion of participants meeting both thresholds to provide a range of interpretive perspectives, recognizing the non-clinical nature of our sample.

##### Work ability

The Work Ability single item score (WAI) [30] was used to assess the participants current work ability compared to their best-ever work ability. The respondent rate their current work ability on a scale from 0 to 10, where 0 means they cannot work at all, and 10 means their work ability is at its best. The WAI has been found to have predictive value for the degree of sick leave and HR-QoL among women working in human service organizations [30].

##### Health-related quality of life.

The RAND-36 was used to measure HR-QoL and includes 36 items that measure eight health domains: physical functioning, role limitations due to physical health problems, role limitations due to emotional problems, energy/fatigue, emotional well-being, social functioning, pain, and general health perceptions. An additional item assesses the change in health the last 12 month and is labelled health transition. Each domain is scored on a scale from 0 to 100, with higher scores indicating better health outcomes [31].

##### Physical activity

To assess the frequency and duration of physical activity, we adapted two items from the Physical Activity Questionnaire [32]. The frequency item was rated on a scale from “none of the days” (0) to “often, 5–7 days” (3), while the duration item ranged from 0 (0 min) to 4 (more than 60 min). For analysis purposes, a physical activity factor was created by multiplying the two items. Previous research has demonstrated that self-reports and single response items are reliable and valid measures of physical activity [33, 34].

### Acceptability

Acceptability was assessed across three indicators and summarised with a Red, Amber and Green classification. The indicators were the recruitment rate relative to the prespecified target of fifty participants, adherence defined as completion of the 12 month assessment together with session attendance at 6 and 12 months, and perceived satisfaction with massage measured with three four-point items (needs met, overall satisfaction, and satisfaction with the extent of massage; higher scores indicate greater satisfaction).

For recruitment we defined Green as achieving at least 70% of the target sample, that is 35 or more of 50; Amber as 50 to 69%, that is 25 to 34 of 50; and Red as less than 50%, that is fewer than 25 of 50.

For adherence we defined Green as at least 80% of participants completing the 12-month assessment and attending at least 60% of their planned sessions; Amber as 60 to 79% completing or 40 to 59% attendance; and Red as less than 60% completing or less than 40% attendance. The 60% attendance threshold corresponds to slightly more than half of the sessions available and was chosen pragmatically to reflect a minimally meaningful level of engagement in a voluntary, low intensity workplace programme. Previous massage studies in clinical populations report that only 50 to 60% of participants attend more than half of scheduled sessions, suggesting that this level of adherence is not uncommon [35].

For satisfaction we defined Green as at least 70% of participants rating themselves mostly or very satisfied on at least two of the three items, Amber as 50 to 69%, and Red as less than 50%. Overall acceptability was classified as Green if at least two indicators were Green and none Red, Amber if any indicator was Amber and none Red, and Red if any indicator was Red. These thresholds were set a priori to provide more nuance than a simple yes or no judgement and to guide decisions about whether to proceed and whether modifications would be required.

Qualitative data collection.

Participants provided written comments in response to three open-ended questions: How have you experienced receiving the massage? Have you experienced any impact of the massage on your health? and have you experienced that the massage has affected your work ability?

### Data analysis

In the quantitative analysis, descriptive statistics were used to summarize participant characteristics, background variables, recruitment, adherence, and satisfaction rates. Categorical data were reported as counts and percentages, while continuous variables were presented as means with standard deviations (SD) or medians with interquartile ranges, depending on the data distribution. To evaluate the limited efficacy of the intervention, Linear Mixed Models (LMM) were applied to data collected at baseline, 6 months, and 12 months. LMM was chosen for its ability to handle repeated measures and missing data, with time treated as both a fixed and repeated effect, an unstructured covariance type, and maximum likelihood estimation [36]. A paired sample t-test was conducted to analyze changes in physical activity between baseline and 12 months. In line with recommendations for feasibility studies by Bowen et al. (2009) [20], effect sizes were prioritized to assess limited efficacy and P-values were reported for transparency but were not used to infer significance, as feasibility studies are not powered for hypothesis testing. For each outcome, we reported means, SDs, change scores with 95% confidence intervals (CI), p-values and effect sizes (Cohen’s *d*), interpreted using conventional thresholds where 0.20–0.49 indicates a small effect, 0.50–0.79 a moderate effect, and ≥ 0.80 a large effect.

Limited efficacy was treated as an exploratory within group appraisal under Bowen’s framework. We summarised the pattern across domains rather than any single measure and flagged a limited efficacy signal when at least three target domains showed a Cohen’s d of 0.20 or greater at 6 or 12 months. We considered durability to be supported when these changes were maintained or further improved at 12 months. These parameters describe feasibility stage signals and are not hypothesis tests or pass or fail rules. To aid interpretation at the individual level, MCID are reported in the where validated thresholds exis**t.** Validated cut-offs have been reported in the literature for PHQ-9, GAD-7 and ISI. No established MCID exists for the PSS-10 or the WAI. For RAND 36, suggested MCID vary by population and are not universally agreed upon [37]. Therefore, no formal MCID thresholds were applied for RAND-36 in this study. All statistical analyses were performed using IBM SPSS version 25.0.

To explore satisfaction with the massage, the open-ended responses were analysed using Braun and Clarke’s six-phase qualitative thematic analysis with an inductive and semantic approach (Braun & Clarke, 2006). This inductive approach allowed themes to emerge directly from the data, rather than testing existing hypotheses or validating previous findings. As a result, the themes were not derived from the questionnaire’s headings. To ensure credibility, two authors (PJ and MW) collaboratively reviewed and discussed the emerging themes before finalizing them. An iterative process was used to compare and refine the coding. In the final step, the authors discussed, revised, and confirmed the themes (see coding schedule, Table 4). Throughout the analysis, potential alternative explanations were considered. Trustworthiness was further established through the transparency of quotations and maintaining an audit trail of the analysis process. Quantitative and qualitative data were integrated during analysis by comparing themes and categories with statistical outcomes to explore consistencies and discrepancies. This integration is further elaborated in the discussion section, where both strands are merged to provide a comprehensive understanding of the intervention’s impact.

## Results

### Study population

Thirty-four nurses registered interest and met the inclusion criteria at screening. Twenty-five attended the consent visit, provided written informed consent, completed baseline measures, and were included in the study. Nine eligible registrants did not attend the visit. No information was collected from the nine individuals who did not attend, as informed consent was provided at the time of the appointment in accordance with ethical guidelines.

**Fig. 1:**
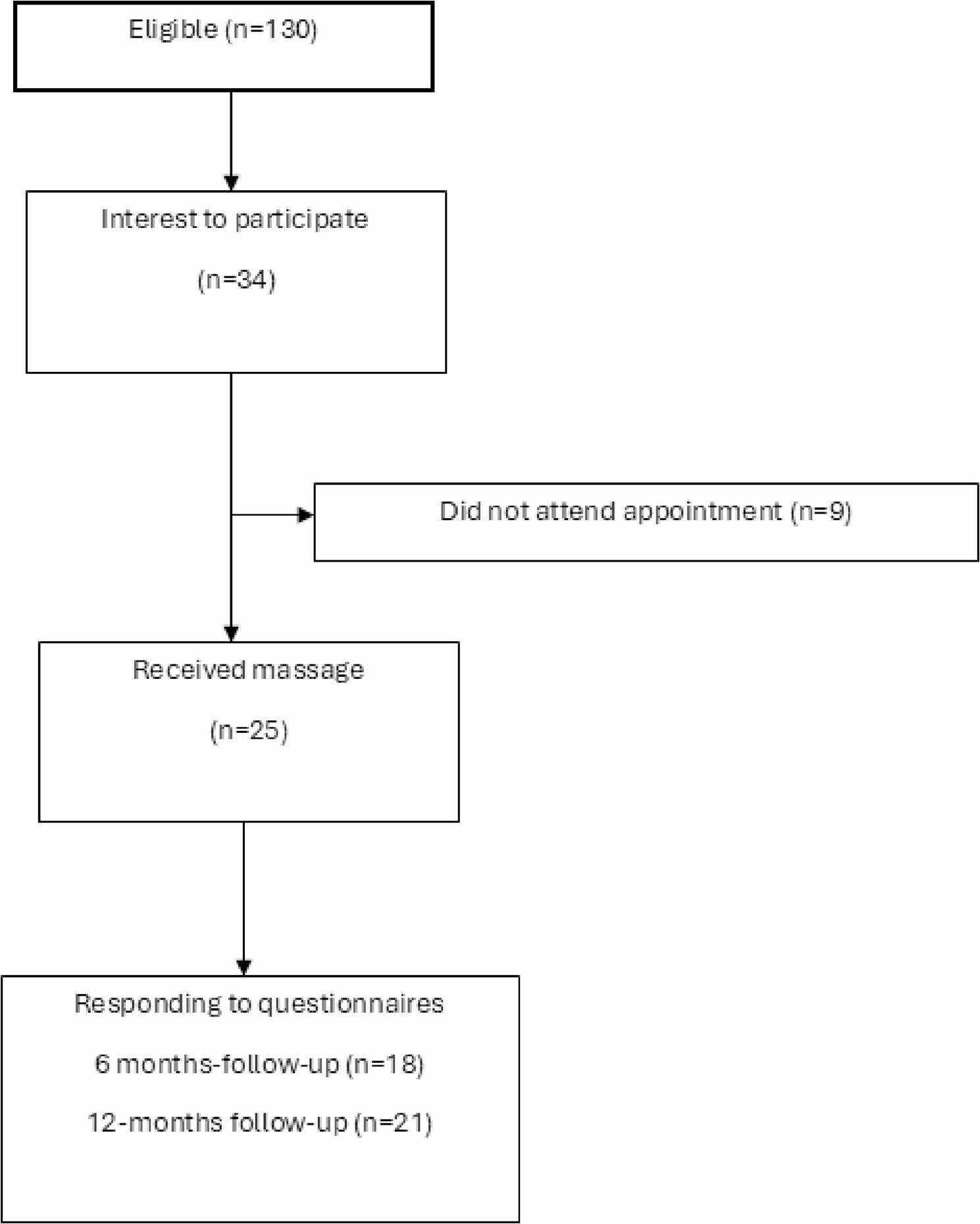
Flowchart of the feasibility study

The participant flow is shown in Fig. 1. The characteristics of participants are presented in Table 1. The mean age was 40 years (SD 11) and 100% were female (*n* = 25). The mean value for the number of years working in healthcare were 11 years (SD 12). The number of participants that reported at least one comorbidity was 5 (20%).

**Table 1:** Characteristics of the participants (n=25)

	Participants n=25
Gender, n (%)	
Female	25 (100)
Age (years), mean (SD)	40 (11)
Years working in healthcare	
Mean (SD)	11 (10.5)
Smoking, n (%)	
Never	10 (40)
Ex-Smokers	11 (44)
Smokers	4 (16)
Tobak uses other than smoking, n (%)	
Never	21(84)
Tobak user	4 (16)
Alcohol, n (%)	
0-1 units per week	14 (56)
1-4 units per week	11 (44)
5-9 units per week	0 (0)
10-14 units per week	0 (0)
15 or more units per week	0 (0)
Diseases, n (%)	
Hypertension	1 (4)
Diabetes	1 (4)
Pulmonary disease	1 (4)
Previous Cancer	2 (8)
Psychiatric disorder	9 (36)
Medicine prescribed, n (%)	
Never	5 (20)
Have medicine prescribed	20 (80)

### Limited efficacy

Results are interpreted against our pre specified multi domain appraisal of limited efficacy. We flag a limited efficacy signal when at least three target domains show a Cohen’s d of 0.20 or greater at 6 or 12 months, and we consider durability to be supported when changes are maintained or further improved at 12 months. MCID are reported as exploratory context to aid interpretation.

#### Stress

**Fig. 2:**
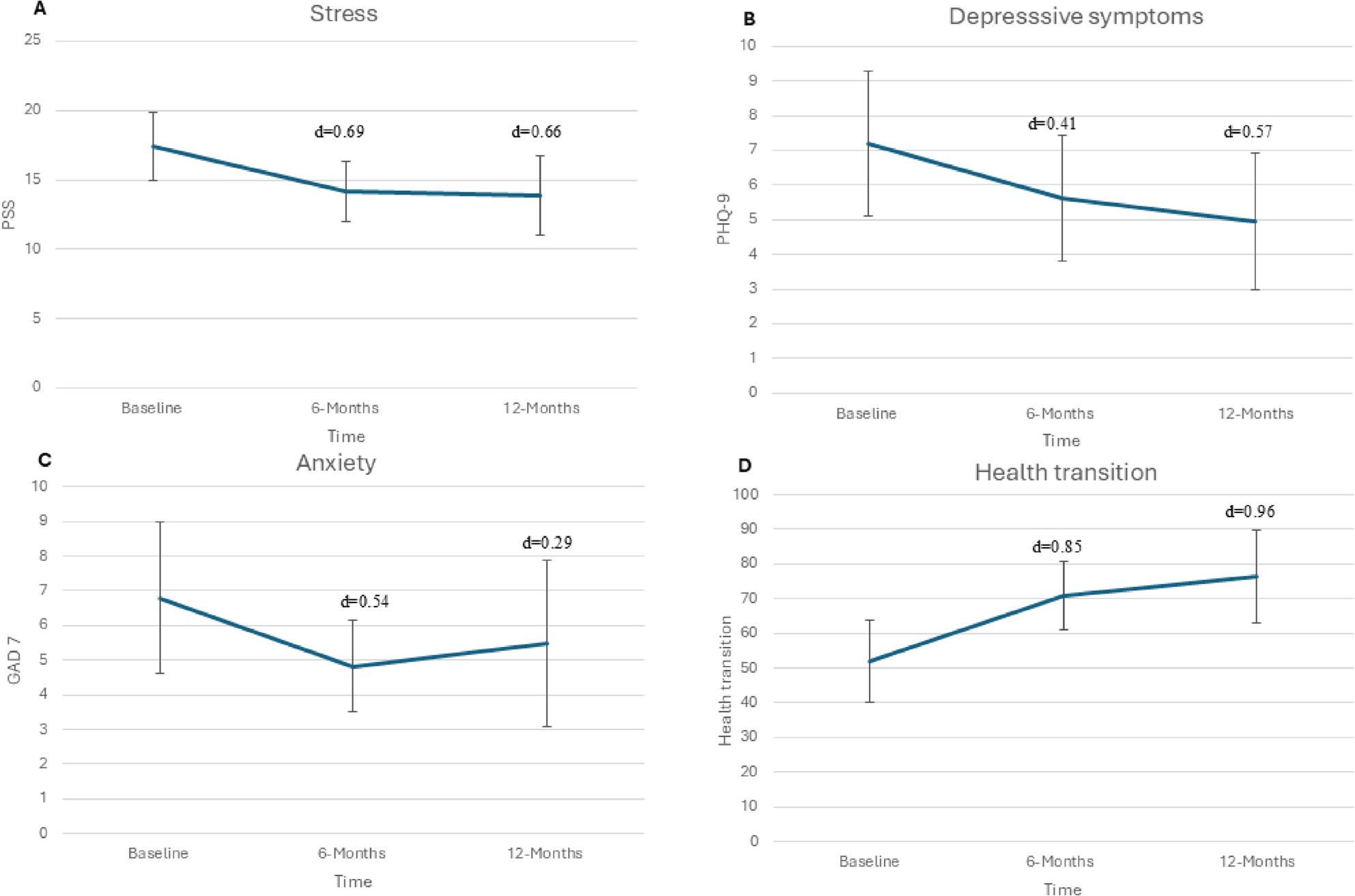
Describes the changes and effects sizes (Cohens d) in stress (**A**), depressive symptoms (**B**), anxiety (**C**) and health transition (**D**) from baseline to 6- and 12 months follow-up

Stress, measured using the PSS-10, showed a moderate reduction at both 6 and 12 months compared to baseline, with effect sizes of d = 0.69 and d = 0.66, respectively (Table 2; Fig. 2A).

**Table 2:** Results of mixed linear model on the outcomes measuring stress, mental health, sleeping problems and work ability

	Time	Estimate	95% CI		Mean (SD)	Effect sizeCohen's d	p	N
Stress								
PSS-10	Baseline(week 0)	0^a^	^-^	^- ^	^17.4 (4.9) ^	^-^	^-^	25
	Follow-up 6 months	-3.330	-5.721	-.938	14.2 (4.2)	.69	.008	18
	Follow-up 12 months	-3.398	-6.236	-.559	13.9 (5.7)	.66	.021	21
Mental Health								
PHQ-9	Baseline (week 0)	0^a^	-	-	7.2 (4.1)	-	-	25
	Follow-up 6 months	-1.583	-3.129	-.036	5.6 (3.6)	.41	.045	18
	Follow-up 12 months	-2.122	-3.917	-.326	4.9 (3.9)	.57	.023	21
GAD-7	Baseline (week 0)	0^a^	-	-	6.8 (4.3)	-	-	25
	Follow-up 6 months	-2.131	-4.380	.118	4.8 (2.6)	.54	.062	18
	Follow-up 12 months	-1.328	-4.013	1.357	5.5 (4.8)	.29	.318	21
KEDS	Baseline (week 0)	0^a^	-	-	16.5 (9.2)	-	-	25
	Follow-up 6 months	-2.463	-5.449	.523	14.7 (8.4)	.20	.101	18
	Follow-up 12 months	-4.093	-7.503	-.683	12.7 (8.5)	.43	.021	21
Sleeping problems								
ISI	Baseline (week 0)	0^a^	-	-	11.8 (4.6)	-	-	25
	Follow-up 6 months	-.535	-3.190	2.120	11.7 (6.2)	.02	.678	18
	Follow-up 12 months	-1.453	-4.174	1.268	10.5 (6.6)	.23	.280	21
Work ability								
WAI	Baseline (week 0)	0^a^	-	-	7.5 (1.5)	-	-	25
	Follow-up 6 months	.588	-.246	1.422	8.0 (1.8)	.31	.157	18
	Follow-up 12 months	.909	.435	1.383	8.5 (1.5)	.67	<.001	21

*Abbreviations*:* GAD-7 *General Anxiety Disorder Scale-7,* ISI *Insomnia Severity Index,* KEDS *Karolinska Exhaustion Disorder Scale,* PHQ-9 *Patient Health Questionnaire-9,* PSS-10 *Perceived Stress Scale-10,* WAI *Work Ability single item score

#### Mental health and sleep

Depressive symptoms, measured by the PHQ-9 total score, showed a small reduction at 6 months (d = 0.41) and a moderate reduction at 12 months (d = 0.57) (Table 2; Fig. 2B). Anxiety, measured with the GAD-7, showed a moderate reduction at 6 months (d = 0.54), but this effect decreased to small at 12 months (d = 0.29) (Table 2; Fig. 2C). Exhaustion, measured by the KEDS, showed a slight increase in effect over time but remained small at both 6 and 12 months (d = 0.20 and d = 0.43). Insomnia, measured by the ISI, showed no effect at 6 months (d = 0.02) and only a small effect at 12 months (d = 0.23) (Table 2).

#### Health-related quality of life

The single-item Health Transition measure showed a large improvement at both 6 months (d = 0.85) and 12 months (d = 0.96) (Table 3; Fig. 2D). Physical functioning showed a moderate improvement at 6 months (d = 0.56), which decreased to small at 12 months (d = 0.29). Role limitations due to physical health showed small improvements at both 6 and 12 months (d = 0.23 and d = 0.29, respectively). A similar pattern was observed for role limitations due to emotional problems, with small improvements at 6 months (d = 0.26) and 12 months (d = 0.23).

**Table 3:** Results of mixed linear model on the outcome health-related quality of life as measured with RAND-36

	Time	Estimate	95 % CI		Mean (SD)	Effect sizeCohen's d	p	N
Physical Functioning.	Baseline (week 0)	0^a^	-	-	83.0 (18.2)	-	-	25
	Follow-up 6 months	8.437	3.389	13.485	91.7 (10.5)	.56	.002	18
	Follow-up 12 months	3.292	-1.764	8.348	86.3 (17.1)	.19	.191	21
Role limitations due to physical health problems	Baseline (week 0)	0^a^	-	-	69.0 (39.7)	-	-	25
	Follow-up 6 months	10.407	-5.331	26.144	77.8 (35.2)	.23	.185	18
	Follow-up 12 months	10.401	-5.728	26.530	79.7 (34.1)	.29	.195	21
Role limitations due to emotional problems	Baseline (week 0)	0^a^	-	-	64.0 (40.7)	-	-	25
	Follow-up 6 months	10.693	-6.810	28.196	74.0 (35.3)	.26	.218	18
	Follow-up 12 months	7.739	-11.168	26.645	73.0 (37.4)	.23	.406	21
Energy/fatigue	Baseline (week 0)	0^a^	-	-	46.2 (20.6)	-	-	25
	Follow-up 6 months	11.885	4.228	19.542	56.1 (22.0)	.47	.004	18
	Follow-up 12 months	8.527	-.212	17.265	55 (23.2)	.40	.055	21
Emotional well-being	Baseline (week 0)	0^a^	-	-	65.9 (11.3)	-	-	25
	Follow-up 6 months	6.534	.774	12.294	72.7 (13.0)	.57	.028	18
	Follow-up 12 months	6.086	-.746	12.918	72.4 (14.3)	.51	.078	21
Social Functioning.	Baseline (week 0)	0^a^	-	-	70.5 (21.3)	-	-	25
	Follow-up 6 months	3.549	-6.911	14.008	73.6 (20.5)	.15	.487	18
	Follow-up 12 months	-1.430	-13.689	10.829	69.6 (26.9)	.04	.811	21
Pain	Baseline (week 0)	0^a^	-	-	59.7 (20.8)	-	-	25
	Follow-up 6 months	11.405	1.959	20.851	70.6 (19.9)	.53	.020	18
	Follow-up 12 months	3.354	-7.201	13.910	63.4 (26.0)	.16	.517	21
General health perception	Baseline (week 0)	0^a^	-	-	58.6 (16.2)	-	-	25
	Follow-up 6 months	6.699	-1.922	15.320	65.6 (19.5)	.40	.121	18
	Follow-up 12 months	4.365	-3.932	12.662	63.3 (20.8)	.26	.287	21
Health transition	Baseline (week 0)	0^a^	-	-	52.0 (23.8)	-	-	25
	Follow-up 6 months	22.814	12.308	33.321	70.8 (19.6)	.85	<.001	18
	Follow-up 12 months	24.876	8.660	41.092	76.3 (26.9)	.96	.004	21

For the energy/fatigue domain, improvements were slightly higher but still considered small at both 6 months (d = 0.47) and 12 months (d = 0.40). In the domain of emotional well-being, the effects were moderate at both 6 and 12 months (d = 0.57 and d = 0.51, respectively). Effect sizes for social functioning were minimal at both 6 months (d = 0.15) and 12 months (d = 0.04). Pain showed a moderate improvement at 6 months (d = 0.53), but the effect decreased to small at 12 months (d = 0.26). A similar trend was observed for general health perceptions, where a small effect at 6 months (d = 0.40) further declined at 12 months (d = 0.26).

#### Work ability

**Fig. 3:**
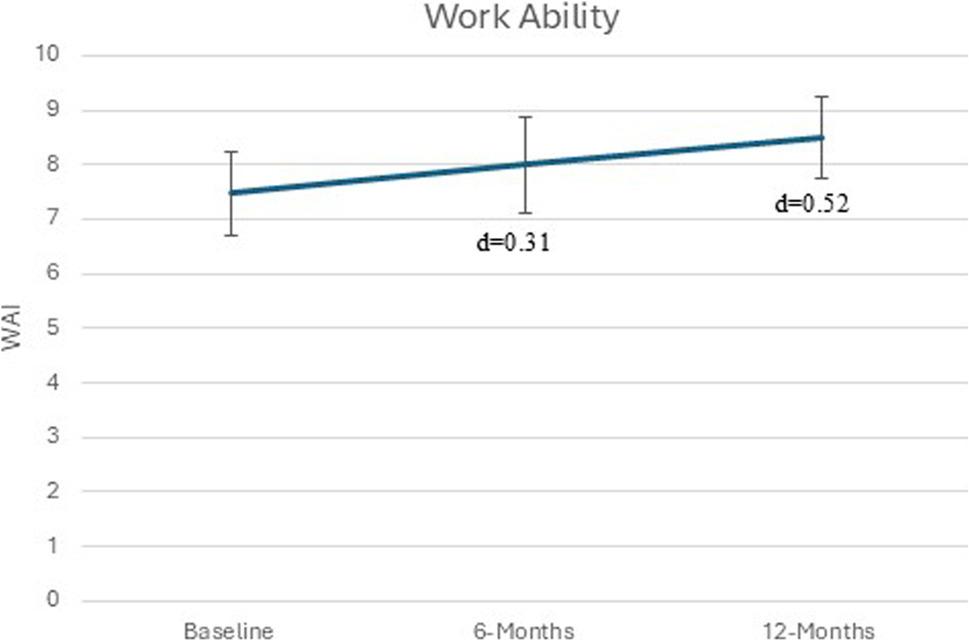
Describes the changes effects sizes (Cohens d) in work ability from baseline to 6- and 12 months follow-up

Work ability, measured with the WAI, showed a small improvement at 6 months (d = 0.31), but the effect increased to moderate at the 12-month follow-up (d = 0.52), indicating higher perceived work ability over time (Table 2; Fig. 3).

#### Physical activity

The mean physical activity component score at baseline was 14.2 (SD = 11.2), increasing to 25.3 (SD = 12.8) after 12 months, corresponding to a large effect (d = 0.92).

#### Summary of limited efficacy

At 6 months, five of seven target domains met the Cohen’s d threshold of 0.20 or greater, namely stress, mental health, pain, HR-QoL and work ability; sleep did not meet the threshold at this time point, and physical activity was assessed at 12 months only. Within HR-QoL, the following subscales were at or above 0.20 at 6 months: health transition, physical functioning, role limitations due to physical health, role limitations due to emotional problems, energy or fatigue, emotional wellbeing, pain, and general health; social functioning was below the threshold (d = 0.15). By 12 months, all seven target domains met or exceeded the 0.20 threshold, including sleep and physical activity. Within HR-QoL, the same subscales remained at or above 0.20 at 12 months, whereas social functioning remained below (d = 0.04). Several subscales showed maintenance or attenuation from 6 to 12 months, for example physical functioning and pain decreased while remaining above 0.20, energy or fatigue and emotional wellbeing remained in the small to moderate range, and health transition increased further. Proportions meeting published MCID for PHQ 9, GAD 7, and ISI are reported in Table [5] and provide individual level context that aligns with the direction of change.

### Acceptability

#### Recruitment

Thirty-four nurses registered interest and 25 enrolled, which corresponds to 50% of the prespecified target of fifty (25 of 50). For context, this equals 19% of the one hundred and thirty eligible nurses. Under the predefined thresholds this corresponds to Amber for recruitment.

#### Adherence

No participant actively discontinued the intervention. Four participants, that is 16%, did not return the 12-month questionnaire and were considered lost to follow up for outcome data. Thus 21 of 25 participants (84%) completed both the intervention and the final data collection, meeting the predefined adherence criterion. By 6 months participants had received a median of 5 sessions, range 2 to 6, reflecting routine scheduling constraints and the planned flexibility to reschedule. For most participants no sessions occurred in June and July due to vacations, with sessions moved to adjacent months. The total number of sessions by twelve months ranged from 9 to 12 most received 10, and two participants received 12 by rescheduling into other months. Thus, some participants front loaded or back loaded sessions within the year while remaining within the planned exposure window. According to the a priori definition, 21 of 25, that is 84%, met the adherence criterion at twelve months which corresponds to Green for adherence.

#### Satisfaction

Perceived satisfaction with massage was high. For needs met, 67% were mostly satisfied and 33% very satisfied. Overall satisfaction was 47% mostly satisfied and 48% very satisfied, with 5% indifferent or somewhat dissatisfied. For satisfaction with the extent of massage, 48% were mostly satisfied and 52% very satisfied. On the composite criterion of at least two of three items rated mostly or very satisfied, at least 95% met the threshold. Under the predefined thresholds, satisfaction corresponds to Green.

#### Overall acceptability

Combining indicators, recruitment was rated Amber, adherence was rated Green, and satisfaction was rated Green, yielding an overall acceptability rating of Green according to the prespecified rule (at least two Green and none Red). This supports feasibility while indicating that recruitment procedures require modification in a future randomised evaluation.

### Qualitative analysis

In total 21 participants provided written comments on three open-ended questions. Three themes were identified in the analysis (a) Positive bodily changes, (b) A valued moment for oneself, and (c) Restoration and ability to cope. Themes and categories with examples of meaning units are presented in Table 4.

**Table 4:** Results of the qualitative thematic analysis

Theme	Quotes
Positive bodily changes	“I’m no longer as tense in my body and can carry out work tasks more easily without getting back pain.” (Participant 101)“I’ve had less pain in my shoulders and neck thanks to regular massage… I can avoid taking painkillers as often.” (Participant 110)
A valued moment for oneself	“I found it very relaxing… 30 minutes just for myself where I could unwind and recover.” (Participant 106) “For me, it's been a small moment each month of some 'me time', relaxation and a bit of relief for my back, which is a plus for me." (Participant 122) "..and was able to relax and forget all the problems from both my private life and work." (Participant 109)
Restoration and ability to cope	“Afterwards I felt much better, both physically and mentally… I was better able to handle work.” (Participant 125) “.. the pain disappears after the massage. I manage my work tasks better and can handle stressful situations in a better way.” (Participant 116)

#### Positive bodily changes

This theme captures the embodied effects of the massage intervention. Participants described how the sessions eased muscular tension and reduced stiffness and pain—particularly in the back, neck, and shoulders. These bodily changes were not only experienced as physical relief, but also as enabling factors in everyday functioning. Some participants reflected on how the massage made it easier to perform work-related tasks or engage in physical activity without discomfort. There were also accounts of increased bodily awareness, where participants became more attentive to posture and physical strain at work. For a few, this sense of physical ease was linked to a decreased need for pain medication or the ability to avoid calling in sick suggesting that even subtle shifts in pain and tension could have meaningful consequences for sustaining work participation.

*“I’ve had less pain in my shoulders and neck thanks to regular massage. I don’t know if my work ability has improved directly*,* but I’ve been able to avoid taking painkillers as often and that’s helped me stay at work instead of going on sick leave because of pain.”* Participant 110.

#### A valued moment for oneself

This theme captures how participants experienced the massage sessions as a rare opportunity to pause, reflect, and focus on themselves. Many situated this experience within the context of a demanding work life, where caregiving roles often left little room for personal recovery. Massage created a distinct space both physically and mentally where they could let go of responsibilities, feel attended to, and regain a sense of self. Participants emphasized the calming atmosphere, the respectful interaction with the therapist, and the significance of being given time without demands. For some, this created not only a sense of emotional relief, but also something to look forward to: a recurring, meaningful event that punctuated the pressures of everyday life.

*“It was very nice to have 30 minutes just for myself*,* where I could relax and recover without needing to do anything for anyone else.”* Participant 106.

#### Restoration and ability to cope

The experience of restoration and ability to cope emerged as a central thread across participants’ reflections. Massage was often described as helping to.

reset after emotionally or physically intense workdays. This sense of restoration involved more than momentary relaxation it contributed to regaining mental clarity, emotional resilience, and physical stamina. Participants noted feeling calmer, more present, or more capable of handling stress after sessions. While the effects were sometimes described as subtle or difficult to isolate, massage was nonetheless integrated into how several participants understood their own ability to cope. In some cases, the anticipation of upcoming massage itself seemed to function as a buffer against the strain of the job. The theme reflects how recovery is not just a result of reduced symptoms, but a deeper process of sustaining oneself in a demanding environment.

*“Given how intense the past six months have been at work*,* I honestly don’t think I would’ve coped as well without the massage. I always looked forward to it and afterwards*,* I felt better both physically and mentally.”* Participant 125.

### Integration of findings

To enhance the interpretation of the intervention’s observed changes, we integrated quantitative outcomes with participants’ qualitative reflections (Table 5).

**Table 5:** Integration of quantitative and qualitative findings

Quantitative outcome	Effect size (Cohen’s d) and % meeting MCID at 12 months	Related qualitative theme	Illustrative interpretation
Perceived stress (PSS-10)	Moderate (d = 0.66)	Restoration and capacity to cope	Participants described feeling mentally restored and better able to manage stress after massage.
Depressive symptoms (PHQ-9)	Moderate (d = 0.57) MCID criterion ≥20% reduction 52 % (n=13)	A valued moment for oneself	Massage was framed as emotionally meaningful and relieving – a moment for self-care amidst demands.
Anxiety(GAD-7)	Small (d = 0.29)MCID criterion 4-point reduction 24 % (n=6)	A valued moment for oneself	Participants described feeling calmer and less emotionally overwhelmed during and after the sessions.
Work ability (WAI)	Moderate (d = 0.52)	Positive bodily changes	Reduced pain and tension supported participants' physical function and ability to manage job demands.
Pain (RAND-36)	Small (d = 0.26)	Positive bodily changes	Participants linked massage to reduced physical discomfort and in some cases reduced medication use or sick leave.
Health transition (RAND)	Large (d = 0.96)	Restoration and capacity to cope	Participants expressed an overall improvement in how they felt and coped, compared to one year earlier.
Emotional well-being (RAND)	Moderate (d = 0.51)	Restoration and capacity to cope	The sense of being emotionally recharged and present after massage was a recurring experience.
Insomnia (ISI)	Small (d=0.23) Liberal MCID criterion≥2.5-point reduction 32% (n=8) Conservative MCID criterion≥6-point reduction 20 % (n=5)	Restoration and capacity to cope	Not explicitly mentioned, participants described feeling better both physically and mentally afterwardsNot explicitly mentioned,
Physical activity (self-report)	Large (d = 0.92)		Not explicitly mentioned, but improved physical ease may have facilitated more movement.

For example, reductions in perceived stress (PSS-10, *d* = 0.66) and improvements in emotional well-being (RAND, *d* = 0.51) and health transition (*d* = 0.96) aligned with participants’ descriptions of massage as a restorative experience that helped them decompress mentally and emotionally. Likewise, improved work ability (*d* = 0.52) and reduced pain (*d* = 0.26) were mirrored in accounts of less tension and increased physical capacity. Although insomnia (ISI) showed only small effect sizes (*d* = 0.23), a meaningful subset of participants reached the MCID threshold: 32% met the liberal (≥ 2.5 points) and 20% the conservative (≥ 6 points) criterion. While sleep improvements were not explicitly mentioned in the qualitative data, descriptions of feeling better physically and mentally may reflect indirect benefits.

Improvements in depressive symptoms (*d* = 0.57) and anxiety (*d* = 0.29) were supported by narratives describing massage as an emotionally meaningful break and a source of calm. Clinically meaningful change was reached by 52% (PHQ-9, MCID ≥ 20% reduction) and 24% (GAD-7, MCID ≥ 4-point reduction) of participants. Physical activity showed a large effect size (*d* = 0.92), though not directly mentioned in free-text responses. However, participants increased bodily ease may have facilitated more movement.

Together, these integrated findings suggest coherence between subjective experience and measured change across several domains, supporting both the plausibility and perceived value of the intervention.

## Discussion

This feasibility study evaluated both the acceptability and limited efficacy of delivering monthly classic massage over one year to nurses. Using predefined feasibility criteria, adherence (84%) and satisfaction (95%) reached the “Green” threshold, indicating acceptability, while recruitment achieved only 50% of the target, rated “Amber.” According to our predefined criteria, limited efficacy was achieved, with at least three target domains showing an effect size of 0.20 or greater at both 6 and 12 months. Many improvements were already evident at 6 months, particularly in stress, mental health, work ability and HR-QoL. Integration with qualitative findings strengthened interpretation: participants described reduced physical tension, valued personal time and a sense of restoration that aligned with observed improvements in stress, emotional well-being and work ability. However, some outcomes, such as sleep and sustained pain relief, showed weaker or inconsistent changes, and anxiety improvements were temporary. These mixed patterns underscore that while classic massage was well tolerated and associated with positive changes in several domains, modifications in recruitment strategies and intervention scope will be important for a future trial.

### Acceptability

Based on our predefined traffic light criteria, two of the three feasibility indicators reached the “Green” threshold. Adherence (84%) and satisfaction (95%) were high, indicating that the intervention was both acceptable and manageable over the one-year period, while recruitment reached only 50% of the target and was therefore rated “Amber.” Together, these findings suggest that the format was feasible and well tolerated but that recruitment procedures require modification in a future trial.

The target of 50 participants was pragmatically set based on financial and organizational constraints specifically, However, we achieved only 50% of the prespecified target, corresponding to 19% of eligible nurses, and was rated “Amber”. The limited one-month recruitment window, absence of reminders, and reliance on a single-option intervention likely contributed to this shortfall. As no data were collected from eligible individuals who chose not to participate, the exact reasons for non-enrolment remain unknown. Prior research shows that HCWs differ in their preferences for wellness interventions [38, 39]. It is plausible that some found a one-year massage program incompatible with personal or work demands. Such incompatibility likely reflects the realities of a changing life situation rather than limitations of the intervention itself. Over a year, factors such as workload, family responsibilities, or health may shift, influencing both participation and perceived benefit. These contextual variations should be considered when planning future trials and may inform more flexible recruitment and delivery strategies, including extended recruitment periods, repeated invitations, and offering participants a choice between massage and another evidence-based stress-reduction option to enhance appeal and generalisability [13, 16]. However, given that recruitment did not meet the predefined threshold, progression to a larger trial in this exact format is not warranted. To reflect real-world conditions, no additional adherence-enhancing strategies were implemented in this feasibility stage; instead, the intervention was delivered under realistic, low-resource conditions reflecting how such a programme might function if offered as part of routine workplace wellness initiatives. With the proposed modifications to recruitment strategies and intervention design, the feasibility criteria could plausibly be met in a future evaluation.

### Limited efficacy

According to our predefined criteria, limited efficacy was achieved, with at least three target domains showing an effect size of 0.20 or greater at both 6 and 12 months. At 6 months, five of seven target domains met this threshold, and by twelve months all seven domains exceeded it, including sleep and physical activity. These findings indicate that the intervention produced consistent within-group improvements across multiple outcomes, fulfilling the limited efficacy criterion. As the study was not powered to assess dose–response relationships, no formal analyses were performed. However, since most participants received a similar number of sessions, it is unlikely that small variations in treatment frequency influenced the observed outcomes.

Although our original hypothesis was that a longer intervention period than the 4–5 weeks used in previous classic massage studies [17–19] would be necessary to influence the broader set of outcomes assessed here, we selected a 12-month duration to maximise the opportunity for change and to observe the sustainability of any effects. We also included a 6-month assessment to determine whether changes could be detected earlier. The finding that many improvements were already evident at 6 months supports the rationale for extending the intervention well beyond the very short durations used in previous trials, while also suggesting that for some domains, a 6-month programme may be sufficient to achieve meaningful change. However, because no earlier measurements were taken, it remains unclear when these changes first emerged during the initial months of the programme. In contrast, other outcomes such as depressive symptoms and exhaustion continued to improve between 6 and 12 months, indicating that a longer duration may be beneficial for these aspects. For a future trial, these patterns suggest testing both shorter and longer delivery periods to identify the minimum effective duration for different outcomes, balancing intervention intensity with feasibility and participant burden. More frequent assessments would also help clarify the onset and progression of change and determine whether early improvements are maintained over time.

Stress, depressive symptoms, exhaustion and anxiety together provided a strong contribution to the overall limited efficacy signal. All four domains exceeded the 0.20 effect size threshold at both 6 and 12 months, except for anxiety at twelve months. Stress reduction was the most consistent change, with moderate effect sizes at both time points (d = 0.69 and 0.66), suggesting that this domain may be particularly responsive to a low-intensity physical relaxation strategy such as classic massage. Depressive symptoms and exhaustion (an aspect of burnout) improved modestly at 6 months and more strongly at 12 months, a parallel trajectory that is consistent with previous research showing a close association between these two outcomes in nurses [40]. This linkage may reflect overlapping mechanisms such as prolonged stress exposure, reduced psychological resources, and physical fatigue, which can be addressed simultaneously by interventions that promote relaxation and restoration. Anxiety showed a moderate improvement at 6 months that declined to a small effect at 12 months, a pattern that may reflect the more variable and context-dependent nature of anxiety symptoms, or a diminishing effect of massage on this domain over time. This suggests that anxiety may require additional or more targeted psychological components in a future trial, while stress-related outcomes might be more amenable to sustained improvement through physical relaxation strategies alone. Since the instruments measuring depression, exhaustion and anxiety reflect symptoms over the past two weeks, and the timing of questionnaire completion was not standardised in relation to participants’ most recent massage session, some variability may have occurred in whether responses reflected general mental health or short-term effects of massage. These diverging trajectories highlight the importance of differentiating psychological outcomes when evaluating intervention impact and may inform future research designs, for instance, by testing combined interventions.

According to our predefined criteria, improvements in HR-QoL and work ability also contributed to the overall limited efficacy signal, with both domains exceeding the 0.20 effect size threshold at 6 and 12 months. HR-QoL improvements were particularly evident in emotional well-being, vitality, physical function and health transition, while work ability increased from a small to a moderate effect over the study period. These changes align with the feasibility aim of detecting multi-domain signals within a low-intensity intervention and support the potential inclusion of these measures in a future trial.

Sleep and pain contributed only partially to the overall limited efficacy signal. Sleep met the 0.20 effect size threshold at twelve months, with 32% of participants reaching the liberal and 20% the conservative MCID threshold for insomnia symptoms. However, these quantitative gains were small, and improved sleep was not reflected in qualitative responses. This suggests that massage alone may not sufficiently address sleep difficulties, which are often multifactorial. Future studies could explore combining massage with interventions more specifically designed to target sleep, such as CBT-I or mindfulness-based approaches. Pain exceeded the 0.20 threshold at 6 months but fell below it at 12 months, despite qualitative accounts of perceived relief. This discrepancy may reflect limitations in the sensitivity of the RAND-36 pain domain or indicate that musculoskeletal pain among HCWs including nurses requires broader strategies such as ergonomic adjustments, physical therapy, and organizational interventions to address underlying causes [41]. Incorporating such components into a future trial could help sustain improvements in pain alongside the other domains contributing to the limited efficacy signal.

### Integration of findings

Integration of quantitative and qualitative findings helped to interpret the limited efficacy signal of exploring both the potential for measurable change and the acceptability of classic massage for nurses. For outcomes such as stress, HR-QoL and work ability, measured improvements were reflected in participants’ descriptions of reduced muscle tension, greater emotional balance and an increased capacity to manage work demands. Themes such as “positive bodily changes,” “a valued moment for oneself,” and “restoration and ability to cope” provided contextual detail, showing how classic massage was experienced not only as symptom relief but also as a resource for sustaining well-being in a demanding clinical environment. These insights strengthen the interpretation of the limited efficacy signal and offer direction for refining outcome measurement. The qualitative accounts also highlighted aspects not captured by the selected instruments, such as the value of anticipation before each session and the experience of protected personal time elements that may help explain the durability of some improvements. For domains like sleep and pain, correspondence between data types was weaker: quantitative gains were modest or inconsistent, while qualitative reflections either downplayed these areas (sleep) or suggested benefits not sustained in scores (pain). These patterns indicate that future trials should consider more sensitive or domain-specific measures and, where relevant, add targeted co-interventions to address outcomes that were less responsive.

### Limitations

Several factors may have influenced the outcomes beyond the massage intervention, including life events occurring near follow-up that could affect participants’ responses. Massage sessions were not always delivered at exact monthly intervals due to rescheduling around vacations and work shifts. Massage sessions were not always delivered at exact monthly intervals due to rescheduling around vacations and work shifts. This flexibility was intentional, as the intervention was designed to reflect real-world delivery and to allow participants to schedule sessions around personal and professional commitments. While such variation may have introduced minor differences in exposure or timing of effects, we believe it likely improved feasibility by reducing attrition and better representing conditions under which a workplace-based wellness programme would operate. Participants also reported relatively favourable mental health and work ability at baseline, increasing the risk of floor and ceiling effects; this reflects our decision not to apply symptom-based inclusion criteria in this feasibility stage. Although this broad inclusion aligns with how massage is often offered in Swedish healthcare, it may have limited the sensitivity of standard outcome measures to detect change. Finally, the study lacked a comparator group, as the primary aim was to assess feasibility and explore whether measurable within-group changes could be detected. For a future trial, we consider an attention-control design preferable to a no-treatment control, as this may provide more meaningful comparisons and reduce attrition among participants allocated to the control condition.

## Conclusion

In this one-group feasibility study, participants showed improvements in stress, mental health, work ability and HR-QoL over 12 months, with several changes evident by 6 months. Adherence and satisfaction indicated high acceptability, while recruitment was below target. The study met predefined limited efficacy criteria, supporting further evaluation in a controlled design. Without a comparator, causality cannot be inferred; future trials should address this and test strategies to improve recruitment and target domains with weaker or inconsistent change, such as sleep and sustained pain relief.

## Data Availability

The datasets used and/or analysed during the current study are available from the corresponding author on reasonable request.

## Abbreviations

GAD-7: General Anxiety Disorder Scale-9
HCW: Healthcare Workers
Hr-QoL: Health-related Quality of Life
ISI: Insomnia Severity Index
KEDS: Karolinska Exhaustion Disorder Scale
LMM: Linear Mixed Models
MCID: Minimum Clinically Important Difference
PSS-10: Perceived Stress Scale-10
PHQ-9: Patient Health Questionnaire-9
WAI: Work Ability single item score

## References

[1] Adriaenssens J, De Gucht V, Maes S. Causes and consequences of occupational stress in emergency nurses, a longitudinal study. J Nurs Manag. 2015;23(3):346–58. 24330154 10.1111/jonm.12138

[2] Maharaj S, Lees T, Lal S. Prevalence and Risk Factors of Depression, Anxiety, and Stress in a Cohort of Australian Nurses. Int J Environ Res Public Health. 2018:16(1). 10.3390/ijerph16010061 PMC6339147 30591627

[3] Adriaenssens J, De Gucht V, Maes S. Determinants and prevalence of burnout in emergency nurses: a systematic review of 25 years of research. Int J Nurs Stud. 2015;52(2):649–61. 25468279 10.1016/j.ijnurstu.2014.11.004

[4] Salvagioni DAJ, Melanda FN, Mesas AE, Gonzalez AD, Gabani FL, Andrade SM. Physical, psychological and occupational consequences of job burnout: A systematic review of prospective studies. PLoS ONE. 2017;12(10):e0185781. 28977041 10.1371/journal.pone.0185781 PMC5627926

[5] Slusher AL, Acevedo EO. Stress induced proinflammatory adaptations: Plausible mechanisms for the link between stress and cardiovascular disease. Front Physiol. 2023;14:1124121. 37007994 10.3389/fphys.2023.1124121 PMC10065149

[6] Jacquier-Bret J, Gorce P. Prevalence of Body Area Work-Related Musculoskeletal Disorders among Healthcare Professionals: A Systematic Review. Int J Environ Res Public Health. 2023;20(1):841. 36613163 10.3390/ijerph20010841 PMC9819551

[7] Occhionero V, Korpinen L, Gobba F. Upper limb musculoskeletal disorders in healthcare personnel. Ergonomics. 2014;57(8):1166–91. 24840049 10.1080/00140139.2014.917205

[8] Sakamoto Y, Amari T, Shimo S. The relationship between pain psychological factors and job stress in rehabilitation workers with or without chronic pain. Work. 2018;61(3):357–65. 30373991 10.3233/WOR-182814

[9] Hayes LJ, O’Brien-Pallas L, Duffield C, Shamian J, Buchan J, Hughes F, Laschinger HK, North N. Nurse turnover: a literature review - an update. Int J Nurs Stud. 2012;49(7):887–905. 22019402 10.1016/j.ijnurstu.2011.10.001

[10] Kakemam E, Kalhor R, Khakdel Z, Khezri A, West S, Visentin D, et al. Occupational stress and cognitive failure of nurses and associations with self‐reported adverse events: A national cross‐sectional survey. J Adv Nurs. 2019;75(12):3609–18. 10.1111/jan.14201 31531990

[11] Toh SG, Ang E, Devi MK. Systematic review on the relationship between the nursing shortage and job satisfaction, stress and burnout levels among nurses in oncology/haematology settings. Int J Evid Based Healthc. 2012;10(2):126–41. 22672602 10.1111/j.1744-1609.2012.00271.x

[12] Försäkringskassan. Analys av skillnader i nyttjande av sjukförsäkringen. Stockholm: Försäkringskassan; 2023. https://www.forsakringskassan.se/download/18.68ee6a4218b7864af031e/1698825778100/analys-av-skillnader-i-nyttjande-av-sjukforsak-delrapport-1-svar-pa-regeringsuppdrag-dnr-fk-2023-002328.pdf.

[13] Catapano P, Cipolla S, Sampogna G, Perris F, Luciano M, Catapano F, Fiorillo A. Organizational and Individual Interventions for Managing Work-Related Stress in Healthcare Professionals: A Systematic Review. Med (Kaunas). 2023;59(10). 10.3390/medicina59101866 PMC10608642 37893584

[14] Barreto DM, Batista MVA. Swedish Massage: A Systematic Review of its Physical and Psychological Benefits. Adv Mind Body Med. 2017;31(2):16–20. 28659510

[15] Zhang M, Murphy B, Cabanilla A, Yidi C. Physical relaxation for occupational stress in healthcare workers: A systematic review and network meta-analysis of randomized controlled trials. J Occup Health. 2021;63(1):e12243. 34235817 10.1002/1348-9585.12243 PMC8263904

[16] Cohen C, Pignata S, Bezak E, Tie M, Childs J. Workplace interventions to improve well-being and reduce burnout for nurses, physicians and allied healthcare professionals: a systematic review. BMJ Open. 2023;13(6):e071203. 37385740 10.1136/bmjopen-2022-071203 PMC10314589

[17] Bost N, Wallis M. The effectiveness of a 15 minute weekly massage in reducing physical and psychological stress in nurses. Aust J Adv Nurs. 2006;23(4):28–33. 16800217

[18] Nazari F, Mirzamohamadi M, Yousefi H. The effect of massage therapy on occupational stress of Intensive Care Unit nurses. Iran J Nurs Midwifery Res. 2015;20(4):508–15. 26257809 10.4103/1735-9066.161001 PMC4525352

[19] Mahdizadeh M, Jaberi AA, Bonabi TN. Massage Therapy in Management of Occupational Stress in Emergency Medical Services Staffs: a Randomized Controlled Trial. Int J Ther Massage Bodyw. 2019;12(1):16–22. PMC6398989 30854151

[20] Bowen DJ, Kreuter M, Spring B, Cofta-Woerpel L, Linnan L, Weiner D, Bakken S, Kaplan CP, Squiers L, Fabrizio C, et al. How we design feasibility studies. Am J Prev Med. 2009;36(5):452–7. 19362699 10.1016/j.amepre.2009.02.002 PMC2859314

[21] Creswell JW, Clark VLP. Designing and conducting mixed methods research: Sage publications; 2017.

[22] Cohen S, Kamarck T, Mermelstein R. A global measure of perceived stress. J Health Soc Behav. 1983;24(4):385–96. 6668417

[23] Nordin S, Palmquist E, Nordin M. Psychometric evaluation and normative data for a Swedish version of the patient health questionnaire 15‐item somatic symptom severity scale. Scand J Psychol. 2013;54(2):112–7. 10.1111/sjop.12029 23294182

[24] Kroenke K, Spitzer RL, Williams JB. The PHQ-9: validity of a brief depression severity measure. J Gen Intern Med. 2001;16(9):606–13. 11556941 10.1046/j.1525-1497.2001.016009606.x PMC1495268

[25] Spitzer RL, Kroenke K, Williams JB, Lowe B. A brief measure for assessing generalized anxiety disorder: the GAD-7. Arch Intern Med. 2006;166(10):1092–7. 16717171 10.1001/archinte.166.10.1092

[26] Besèr A, Sorjonen K, Wahlberg K, Peterson U, Nygren A, Asberg M. Construction and evaluation of a self rating scale for stress-induced exhaustion disorder, the Karolinska Exhaustion Disorder Scale. Scand J Psychol. 2014;55(1):72–82. 24236500 10.1111/sjop.12088 PMC4235404

[27] Toussaint A, Hüsing P, Gumz A, Wingenfeld K, Härter M, Schramm E, Löwe B. Sensitivity to change and minimal clinically important difference of the 7-item Generalized Anxiety Disorder Questionnaire (GAD-7). J Affect Disord. 2020;265:395–401. 32090765 10.1016/j.jad.2020.01.032

[28] Morin CM, Belleville G, Belanger L. Validation of the insomnia severity index. Sleep. 2006;29:A258–9.

[29] Yang M, Morin CM, Schaefer K, Wallenstein GV. Interpreting score differences in the Insomnia Severity Index: using health-related outcomes to define the minimally important difference. Curr Med Res Opin. 2009;25(10):2487–94. 19689221 10.1185/03007990903167415

[30] Ahlstrom L, Grimby-Ekman A, Hagberg M, Dellve L. The work ability index and single-item question: associations with sick leave, symptoms, and health–a prospective study of women on long-term sick leave. Scand J Work Environ Health. 2010;36(5):404–12. 20372766 10.5271/sjweh.2917

[31] Hays RD, Sherbourne CD, Mazel RM. The RAND 36-Item Health Survey 1.0. Health Econ. 1993;2(3):217–27. 8275167 10.1002/hec.4730020305

[32] Craig CL, Marshall AL, Sjostrom M, Bauman AE, Booth ML, Ainsworth BE, Pratt M, Ekelund U, Yngve A, Sallis JF, et al. International physical activity questionnaire: 12-country reliability and validity. Med Sci Sports Exerc. 2003;35(8):1381–95. 12900694 10.1249/01.MSS.0000078924.61453.FB

[33] Jackson AW, Morrow JR Jr., Bowles HR, FitzGerald SJ, Blair SN. Construct validity evidence for single-response items to estimate physical activity levels in large sample studies. Res Q Exerc Sport. 2007;78(2):24–31. 17479571 10.1080/02701367.2007.10599400

[34] Aadahl M, Kjaer M, Kristensen JH, Mollerup B, Jorgensen T. Self-reported physical activity compared with maximal oxygen uptake in adults. Eur J Cardiovasc Prev Rehabil. 2007;14(3):422–8. 17568243 10.1097/HJR.0b013e3280128d00

[35] Nemati D, Hinrichs R, Johnson A, Lauche R, Munk N. Massage Therapy as a Self-Management Strategy for Musculoskeletal Pain and Chronic Conditions: A Systematic Review of Feasibility and Scope. J Integr Complement Med. 2024;30(4):319–35. 37878283 10.1089/jicm.2023.0271

[36] Molenberghs G, Verbeke G. A review on linear mixed models for longitudinal data, possibly subject to dropout. Stat Modelling. 2001;1(4):235–69.

[37] Samsa G, Edelman D, Rothman ML, Williams GR, Lipscomb J, Matchar D. Determining clinically important differences in health status measures: a general approach with illustration to the Health Utilities Index Mark II. PharmacoEconomics. 1999;15(2):141–55. 10351188 10.2165/00019053-199915020-00003

[38] Wu A, Radhakrishnan V, Targan E, Scarella TM, Torous J, Hill KP. Self-Reported Preferences for Help-Seeking and Barriers to Using Mental Health Supports Among Internal Medicine Residents: Exploratory Use of an Econometric Best-Worst Scaling Framework for Gathering Physician Wellness Preferences. JMIR Med Educ. 2021;7(4):e28623. 34612838 10.2196/28623 PMC8529465

[39] Wu A, Parris RS, Scarella TM, Tibbles CD, Torous J, Hill KP. What gets resident physicians stressed and how would they prefer to be supported? A best-worst scaling study. Postgrad Med J. 2022;98(1166):930–5. 34810273 10.1136/postgradmedj-2021-140719

[40] Chen C, Meier ST. Burnout and depression in nurses: A systematic review and meta-analysis. Int J Nurs Stud. 2021;124:104099. 34715576 10.1016/j.ijnurstu.2021.104099

[41] Albanesi B, Piredda M, Bravi M, Bressi F, Gualandi R, Marchetti A, Facchinetti G, Ianni A, Cordella F, Zollo L, et al. Interventions to prevent and reduce work-related musculoskeletal injuries and pain among healthcare professionals. A comprehensive systematic review of the literature. J Saf Res. 2022;82:124–43. 10.1016/j.jsr.2022.05.004 36031239

